# Dynamic Feature Extraction-Based Quadratic Discriminant Analysis for Industrial Process Fault Classification and Diagnosis

**DOI:** 10.3390/e25121664

**Published:** 2023-12-16

**Authors:** Hanqi Li, Mingxing Jia, Zhizhong Mao

**Affiliations:** 1 College of Information Science and Engineering, Northeastern University, Shenyang 110819, China; lihanqi2016@stumail.neu.edu.cn (H.L.); jiamingxing@ise.neu.edu.cn (M.J.); 2 Laboratory of Synthetical Automation for Process Industries, Northeastern University, Shenyang 110819, China

**Keywords:** dynamic process monitoring, discriminant analysis, multivariate statistics, supervised learning, cold rolling mill

## Abstract

This paper introduces a novel method for enhancing fault classification and diagnosis in dynamic nonlinear processes. The method focuses on dynamic feature extraction within multivariate time series data and utilizes dynamic reconstruction errors to augment the feature set. A fault classification procedure is then developed, using the weighted maximum scatter difference (WMSD) dimensionality reduction criterion and quadratic discriminant analysis (QDA) classifier. This method addresses the challenge of high-dimensional, sample-limited fault classification, offering early diagnosis capabilities for online samples with smaller amplitudes than the training set. Validation is conducted using a cold rolling mill simulation model, with performance compared to classical methods like linear discriminant analysis (LDA) and kernel Fisher discriminant analysis (KFD). The results demonstrate the superiority of the proposed method for reliable industrial process monitoring and fault diagnosis.

## 1. Introduction

The continuous development of data acquisition methods has substantially widened the capacity to efficiently accumulate extensive datasets in industrial processes. Data-driven techniques serve a crucial role in improving manufacturing operations [1,2,3,4]. Employing historical data for online process monitoring to prevent critical failures and accidents is a real-world utilization of data-driven techniques, spanning fault detection and diagnosis. Fault detection serves as the initial stage in process monitoring, issuing advance notification of potential process failures. Following this, fault diagnosis identifies the specific type of anomaly detected, offering guidance for subsequent troubleshooting [5,6,7].

Typical multivariate statistical techniques employed for process monitoring and fault detection include principal component analysis (PCA), partial least squares (PLS) and their enhanced variations. These techniques use control charts such as SPE and T-square to evaluate whether the ongoing process is within normal operational bounds. The examination of the contribution of each variable to these statistics aids in the identification of the variables responsible for faults and supports fault diagnosis [8,9,10]. Moreover, research focusing on PCA Bayesian network (PCA-BN) has significantly enriched fault diagnosis methodologies, especially through the use of contribution plots [11,12]. Furthermore, optimized PLS and parity methods have shown progress in nonlinear fault diagnosis [13,14].

However, industrial historical datasets often include both normal and labeled fault data, emphasizing the significance of supervised classification in fault diagnosis tasks [15]. Through supervised classification, each fault data class can be distinguished from others, enabling the allocation of online anomaly data to the relevant fault class. This streamlines the process of implementing targeted solutions [7,16,17].

Linear discriminant analysis (LDA), as a representative supervised classification, is understood through two closely linked interpretations. The first, known as Fisher’s LDA, was initially introduced by Fisher in 1936 for binary classification and later extended to handle multiple classes by Rao [18,19]. The second interpretation is Bayesian LDA, which operates as a linear Gaussian classifier based on Bayes’ rule [20]. These two perspectives are essentially equivalent under specific conditions [21]. In practical conditions, Fisher’s LDA and its refined variants often serve as dimensionality reduction techniques [22], while Bayesian LDA and more generalized Gaussian classifiers are typically employed to establish decision boundaries [23]. In some cases, the linear transformation based on the Fisher criterion and the application of Bayesian linear discriminant function can be seen as sequential stages in a classification task, often referred to as reduced-rank LDA [20].

In situations characterized by high-dimensional datasets with limited samples, the classic Fisher criterion encounters a significant challenge related to the potential singularity of within-class covariance matrices. To tackle this challenge, several enhanced approaches leveraging subspace or null space techniques have been proposed [24,25,26]. However, it is imperative to recognize that such preliminary dimensionality reduction may result in the loss of discriminant information within the non-principal components or non-null spaces, potentially impacting the overall classification performance [27]. While certain discriminant analysis techniques based on matrix exponentials exhibit strong discriminative capabilities, they impose considerable computational resource demands, especially when applied to high-dimensional datasets [28,29]. To maximize the retention of discriminative features, several modified Fisher criteria have been introduced. These criteria are designed to avoid issues related to matrix inversion and null space analysis. Notably, the maximum scatter difference (MSD) and maximum margin criterion (MMC) have emerged successively to address the challenge of small sample size (SSS) problems [30,31,32].

Quadratic discriminant analysis (QDA) is an extension of Bayesian LDA, offering increased flexibility and enhanced nonlinear classification capability [33]. However, its quadratic relationship with feature dimension renders it more susceptible to issues associated with high-dimensional data [34]. To tackle this, various improved methods aim to refine parameter estimation for quadratic discriminant functions, reducing complexity. These methods include regularized discriminant analysis (RDA), which combines the covariance estimation aspects of LDA and QDA and other approaches utilizing sparse estimators, such as sparse QDA (SQDA) [35,36,37]. However, many of these methods necessitate complex assumptions and extensive parameter fine-tuning [34]. In recent years, hybrid techniques combining preliminary dimensionality reduction and feature extraction have been introduced. These include ridge-forward quadratic discriminant (RFQD), envelope discriminant subspace (ENDS) QDA, locally linear embedding (LLE) QDA, PCA-QDA and others [38,39,40,41].

To cope with dynamic and nonlinear scenarios, our proposed method combines QDA with a novel dynamic feature extraction strategy. Our research primarily focuses on optimizing scenarios where online fault magnitudes are relatively smaller than those in historical datasets, with the objective of enhancing early classification capabilities.

This paper presents the following key contributions:

(1) An approach to represent dynamics within multivariate time series data is introduced. It quantifies the dynamic relationships among lag submatrices by reconstructing past samples using current data. The reconstruction errors serve as dynamic features to expand the sample set.
(2) A fault classification procedure for dynamic nonlinear processes is developed. It utilizes the WMSD criterion for dimensionality reduction of fault samples integrated with dynamic features and employs QDA for classification. The effectiveness of this approach is demonstrated in a subsequent novel cold rolling mill simulation case study.

## 2. Related Researches

### 2.1. Fisher Criterion Based Dimensionality Reduction

When referring to the term Fisher’s discriminant analysis (FDA), it typically implies two main aspects: dimensionality reduction and classification. It’s noteworthy that the classification phase shares commonalities with Bayesian LDA, which will be explored in the subsequent subsection. Thus, this section focuses on explaining the dimensionality reduction technique based on the Fisher criterion, which involves the projection of high-dimensional data into a lower-dimensional feature space. This transformation aims to maximize the between-class scatter while minimizing the within-class scatter.

We consider a training set as denoted by $X\in R^{n\times p}$, where *n* represents the number of observations and *p* denotes the number of features. This dataset comprises *K* distinct classes, with $n_{i}$ being the count of observations within the *i*-th class. The sample mean $m_{i}$ for the *i*-th class is calculated as follows:

(1) $$m_{i}=\frac{1}{n_{i}}\sum_{C=i}x_{j}\;i=1,2,\ldots ,K\;j=1,2,\ldots ,n_{i}$$

where $x_{j}$ represents a sample belonging to the *i*-th class.

We employ the covariance of the class means to establish the between-class scatter, denoted as $S_{B}$ and the covariances within distinct classes to define the within-class scatter, represented as $S_{W}$. The expressions for these matrices are as follows:

(2) $$S_{B}=\sum_{i=1}^{K}n_{i}{\left(m_{i}-\bar{m}\right)}^{T}\left(m_{i}-\bar{m}\right)$$

(3) $$S_{i}=\sum_{C=i}{\left(x_{j}-m_{i}\right)}^{T}\left(x_{j}-m_{i}\right)$$

(4) $$S_{W}=\sum_{i=1}^{K}S_{i}$$

where $\bar{m}$ represents the mean of all samples.

The projection from the original dataset to a reduced $p^{\prime }$-dimensional space is expressed as follows:

(5) Y=XW

Here, the resulting matrix $Y\left(n\times p^{\prime }\right)$ denotes the reduced dataset and the projection matrix $W\left(p\times p^{\prime }\right)$ is composed of the weight vectors *w*. This projection helps to reduce dimensionality while preserving critical information.

To obtain the weight vectors, we employ the Fisher criterion, which is formulated as follows:

(6) $$w=\underset{w}{\arg\max}\;\;\frac{w^{T}S_{B}w}{w^{T}S_{W}w}$$

The aim of this optimization is to maximize the ratio of the between-class scatter to the within-class scatter. This ratio is essentially a generalized Rayleigh quotient and the vector that maximizes the function corresponds to the eigenvector associated with the maximum eigenvalue of $S_{W}^{-1}S_{B}$. The eigenvalue decomposition is expressed as:

(7) $$S_{W}^{-1}S_{B}w=\lambda w$$

In this equation, λ represents the eigenvalues and *w* denotes the eigenvectors. The top $p^{\prime }$ eigenvectors collectively form the projection matrix *W*. It’s important to note that the maximum permissible value for $p^{\prime }$, equivalent to the rank of $S_{B}$, is no greater than both *p* and K−1. This constraint ensures that the dimensionality of the target space is suitably low, making it suitable for subsequent classification tasks.

### 2.2. Bayesian Linear and Quadratic Discriminant Analysis

Bayesian discriminant rules are widely employed probability-based classification techniques, encompassing methods such as Bayesian LDA and QDA. In both LDA and QDA, the classification task is fundamentally rooted in the pursuit of the highest posterior probability, which is expressed as:

(8) $$C\left(x\right)=\underset{i}{\arg\max}P\left(C=i|x\right)\;i=1,2,\ldots ,K$$

Guided by Bayesian theory, the posterior probability of sample *x* belonging to class *i* is computed as follows:

(9) $$P\left(C=i|x\right)=\frac{P\left(x|C=i\right)P\left(C=i\right)}{\sum_{k=1}^{K}P\left(x|C=k\right)P\left(C=k\right)}$$

Here, $P\left(x|C=i\right)$ represents the probability density function of the *i*-th class and $P\left(C=i\right)$ denotes the prior probability of the *i*-th class. It’s worth noting that this prior probability signifies the proportion of the sample size within that class relative to the total sample size.

Assuming that the samples of the *i*-th class follow the multivariate normal distribution, whose probability density function is expressed as follows:

(10) $$P\left(x|C=i\right)=\frac{1}{{\left(2\pi \right)}^{p/2}{\left|\Sigma_{i}\right|}^{1/2}}e^{-\frac{1}{2}\left(x-\mu i\right)\Sigma_{i}^{-1}{\left(x-\mu i\right)}^{T}}$$

where $\mu_{i}$ represents the mean of the *i*-th class and $\Sigma_{i}$ denotes the covariance matrix of the *i*-th class.

By substituting Equation (10) into Equation (9) and subsequently applying logarithm, the Bayesian discriminant function is derived. For the LDA classifier, the covariance matrices of all classes are assumed to be equal. The linear discriminant function takes on the following form:

(11) $$\begin{array}{cc} \delta_{i}\left(x\right) & =\log P\left(C=i|x\right)\\ \; & =x\Sigma^{-1}{\mu_{i}}^{T}-\frac{1}{2}\mu_{i}\Sigma^{-1}{\mu_{i}}^{T}+\log P\left(C=i\right) \end{array}$$

In the equation, Σ is commonly referred to as the pooled within-class covariance matrix. Its unbiased estimate corresponds to the weighted average of the covariances from all classes and is computed as follows:

(12) $$\Sigma =\frac{1}{n-K}\sum_{C=i}\left(n_{i}-1\right){\left(x_{j}-\mu_{i}\right)}^{T}\left(x_{j}-\mu_{i}\right)$$

When new observations are substituted to the discriminant functions associated with each class, the class that yields the highest value becomes the output result of the LDA classifier.

In the LDA classifier, distinct classes are separated by hyperplanes. However, when we relax the assumption of equal covariances across all classes, LDA transforms into QDA. In the QDA classifier, different classes are demarcated by quadratic hypersurfaces.

The discriminant function for QDA is expressed as follows:

(13) $$\delta_{i}=-\frac{1}{2}\log\left|\Sigma_{i}\right|-\frac{1}{2}\left(x-\mu_{i}\right)\Sigma_{i}^{-1}{\left(x-\mu_{i}\right)}^{T}+\log P\left(C=i\right)$$

where $\left|\cdot \right|$ represents the determinant.

QDA offers the advantage of non-linear classification, but it comes with significantly higher model complexity compared to LDA. This increased complexity elevates the risks of overfitting and issues related to singular values. Therefore, maintaining sufficient classification information within a reduced dataset is of paramount importance when employing QDA as a classification method.

## 3. Dynamic Feature Extraction Based Quadratic Discriminant Analysis

### 3.1. Dynamic Extraction and Feature Extension

In our prior research [42], we introduced methods for process monitoring and fault detection, which primarily centered on extracting direct dynamic representations from process data. These techniques yielded significant results. In this paper, we extend these methods to the initialization of classified data. More precisely, we utilize the dynamic process information derived from an extensive volume of normal training data to extract valuable features for subsequent fault classification procedures.

For a standardized and whitened normal dataset represented by $X_{0}$ with dimensions n×p, we form its lagged submatrices at a one-step lag as follows:

(14) $$\begin{array}{cc} X_{k-1} & ={\left[x_{1}^{T}\;\;x_{2}^{T}\;\;x_{3}^{T}\;\;\ldots x_{n-1}^{T}\right]}^{T}\\ X_{k} & ={\left[x_{2}^{T}\;\;x_{3}^{T}\;\;x_{4}^{T}\;\;\ldots x_{n}^{T}\right]}^{T} \end{array}$$

Here, each row vector $x_{j}$ represents an individual sample from $X_{0}$, with j=1,2,‣,n.

To establish a direct dynamic representation between these submatrices, our objective is to determine the optimal transformation matrix that relates them. This is achieved by solving the following optimization problem:

(15) $$R=\underset{R}{\arg\min}\;\;∥X_{k}R-X_{k-1}∥$$

The transformation matrix *R* is a square matrix with dimensions p×p and is designed for convenient application to online data. When $X_{k}$ is invertible, the transformation matrix $R=X_{k}^{-1}X_{k-1}$, calculated using matrix inversion, precisely satisfies the requirements of the objective function and minimizes it to zero. However, for more general cases, we use the Moore–Penrose pseudo-inverse, which is also suitable for rank reduction. The calculation of *R* is as follows:

(16) $$R=pinv\left(X_{k}\right)\cdot X_{k-1}$$

Here, pinv(·) denotes the Moore–Penrose pseudo-inverse. Previous research has demonstrated that the reduced-rank version of the pseudo-inverse already contains a sufficient amount of dynamic information. To minimize information redundancy, we conduct a singular value decomposition (SVD) on $X_{k}$, which is expressed as follows:

(17) $$R=V\cdot pinv\left(S\right)\cdot U^{T}\cdot X_{k-1}$$

The reduced-rank version of SVD and the corresponding *R* can be expressed as:

(18) $$X_{k}\approx \tilde{U}\tilde{S}{\tilde{V}}^{T}$$

(19) $$R=\tilde{V}{\tilde{S}}^{-1}{\tilde{U}}^{T}X_{k-1}$$

Here, $\tilde{U}$, $\tilde{S}$, $\tilde{V}$ have dimensions of n×r, r×r and p×r, respectively, with *r* being smaller than both *n* and *p*.

With the aid of the transformation matrix *R*, we can utilize the current submatrix $X_{k}$ to reconstruct the past submatrix $X_{k-1}$, as follows:

(20) $${\tilde{X}}_{k-1}=X_{k}R$$

Moreover, the transformation matrix *R* can be applied to the fault training datasets. We introduce a novel dynamic feature set by incorporating the error of reconstruction. These features are subsequently combined with the original samples to create an augmented vector, serving as the subject of further processing. The computational process can be described as follows:

(21) $$\begin{array}{cc} {\tilde{e}}_{j} & ={\tilde{x}}_{j-1}-x_{j-1}\\ \; & =x_{j}R-x_{j-1} \end{array}$$

(22) $${\tilde{x}}_{j}=\left[x_{j}\;\;{\tilde{e}}_{j}\right]$$

In this context, $x_{j}$ denotes a sample from the *i*th fault training dataset with $n_{i}$ samples. Here, *j* spans from 2 to $n_{i}$. This computation leads to the formation of reconstructed error, denoted as ${\tilde{e}}_{j}$ and the augmented vector, represented as ${\tilde{x}}_{j}$. The augmented vector is a fusion of static and dynamic features.

The dynamic reconstruction procedure described above is adaptable and can be readily applied to either a pair or a series of new online samples.

### 3.2. Improved Dynamic Discriminant Analysis Classifier

To enhance the performance of nonlinear classification, our proposed approach leverages an improved QDA classifier. The effectiveness of QDA is sensitive to the dimensionality of the input data. As a preliminary step, we employ dimensionality reduction based on a modified Fisher criterion.

The traditional Fisher criterion typically employs the inverse matrix of the pooled within-class scatter to compute the transformation matrix. However, this approach carries the risk of singular values, particularly when dealing with datasets with numerous features but limited samples, a case frequently encountered in process fault analysis. Even if we opt for a pseudo-inverse substitution, the utilization of too many parameters can lead to overfitting. In such cases, the model becomes excessively tuned to the training data, making it challenging to promptly detect minor faults.

To address these challenges, this paper adopts the WMSD criterion to formulate a dimensionality reduction model with enhanced generalization capabilities [32]. The WMSD criterion can be expressed as:

(23) $$\begin{array}{cc} w & =\underset{w}{\arg\max}\;\;\theta w^{T}S_{B}w-\left(1-\theta \right)w^{T}S_{W}w\\ \; & =\underset{w}{\arg\max}\frac{w^{T}\left(\theta S_{B}-\left(1-\theta \right)S_{W}\right)w}{w^{T}w}\\ s.t. & \;w^{T}w=1 \end{array}$$

In this expression, 0<θ<1 represents a weight coefficient. Notably, the WMSD criterion replaces $S_{W}^{-1}S_{B}$ from the classic Fisher criterion with $\theta S_{B}-\left(1-\theta \right)S_{W}$. In order to find the optimal value of the new Rayleigh quotient, we apply eigenvalue decomposition to $\theta S_{B}-\left(1-\theta \right)S_{W}$ to obtain the weight vectors *w* and the projection matrix *W*. This substitution not only eliminates the need for matrix inversion but also provides a mechanism for adjusting the weight of the pooled within-class scatter.

While dimensionality reduction based on the modified Fisher criterion effectively constrains the input dimensions of QDA to a maximum of K−1 (where *K* represents the number of classes), it is essential to acknowledge that valuable information might still be sacrificed within this limited features. To enhance the discriminative capabilities of our classifier without increasing the input dimensions for QDA, we expand the input features of the modified Fisher model. We introduce the dynamic reconstructed error as new features to both training and testing samples. The augmented vectors are structured as Equation (22). As a result, the dimensionality model keeps the output dimension unchanged while accommodating more dynamic information that proves advantageous for classification. Such combined techniques are particularly suited for detecting online faults with smaller magnitudes compared to those present in the training sets.

**Figure 1:**
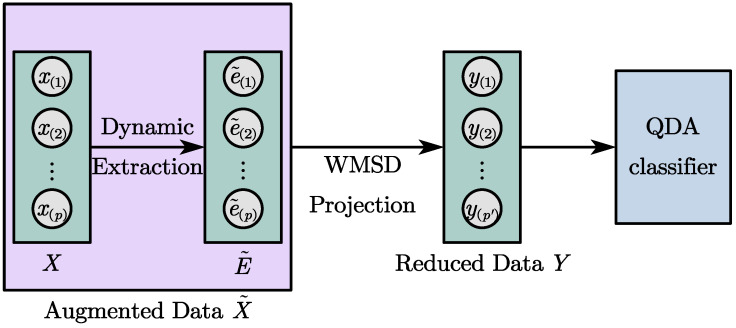
Learning framework of the proposed method.

Figure 1 illustrates the learning framework of the proposed method, delineating the transformation of static and dynamic features across the algorithm’s layers. In this schematic representation, the *p*-dimensional *X* corresponds to the fault training dataset, while matrix $\tilde{E}$ encompasses the reconstruction error vectors. The fusion of dynamic features within $\tilde{E}$ and static features from *X* creates an augmented dataset $\tilde{X}$, which undergoes projection into a $p^{\prime }$-dimensional space guided by the WMSD criterion. This transformation paves the way for the training of the QDA classifier.

### 3.3. Offline Modeling

During the offline modeling stage, the preparation of two distinct training datasets is essential. The first dataset comprises normal data, characterized by the stable operation of the process under study and a substantial sample size. The second dataset consists of fault training data, which encompasses multiple classes, each represented by a significantly smaller sample size compared to the normal data. This discrepancy in sample size accurately mirrors the real-world conditions observed in industrial processes.

In the initial stage, we begin by substituting the preprocessed normal training dataset into Equation (14) and then use Equations (18) and (19) to calculate the transformation matrix, referred to as *R*. Following this, we proceed to initialize the fault training datasets using the baseline of normal data. These fault datasets are subsequently utilized in into Equations (21) and (22) to calculate the augmented vectors $\tilde{x}$ with dynamic features.

Assuming we are dealing with a total of *K* fault classes. In this context, ${\tilde{X}}_{i}$ represents the augmented training dataset specific to the *i*-th fault class. The sample sizes of various classes and the total sample size are represented by $n_{i}$ and *n* respectively. Based on these datasets, we can readily compute the respective class means $m_{i}$ and the overall sample mean $\bar{m}$. From these calculations, we can derive the covariance matrix for each class, subsequently enabling the assessment of within-class and between-class scatter matrices.

(24) $$S_{B}=\sum_{i=1}^{K}n_{i}{\left(m_{i}-\bar{m}\right)}^{T}\left(m_{i}-\bar{m}\right)$$

(25) $$S_{i}=\sum_{C=i}{\left({\tilde{x}}_{j}-m_{i}\right)}^{T}\left({\tilde{x}}_{j}-m_{i}\right)$$

(26) $$S_{W}=\sum_{i=1}^{K}S_{i}$$

where ${\tilde{x}}_{j}$ represents an augmented vector belonging to class *i*.

Subsequently, according to the WMSD criterion expressed as Equation (23), we perform eigenvalue decomposition of $\theta S_{B}-\left(1-\theta \right)S_{W}$ as shown below:

(27) $$(\theta S_{B}-\left(1-\theta \right)S_{W})w=\lambda w$$

To create the projection matrix *W*, we preserve the eigenvectors *w* corresponding to the top $p^{\prime }$ eigenvalues λ, a selection that is determined via cross-validation. The matrix *W* can be succinctly expressed as:

(28) $$W=\left[w_{1}\;\;w_{2}\;\;\ldots \;\;w_{p\prime }\right]$$

Afterward, the low-dimensional projection of various fault classes can be calculated as follows:

(29) $$Y_{i}={\tilde{X}}_{i}W$$

In order to train a QDA classifier, it is essential to determine the mean, covariance matrix and prior probability of each class. These parameters can either be recomputed using the projected data $Y_{i}$ or derived from previously calculated values:

(30) $$\mu_{i}=m_{i}\times W$$

(31) $$\Sigma_{i}=W^{T}\frac{1}{n_{i}}S_{i}W$$

(32) $$P\left(C=i\right)=\frac{n_{i}}{n}$$

In these equations, $\mu_{i}$ and $\Sigma_{i}$ correspond to the mean and covariance matrix of $Y_{i}$ from the *i*-th class, while $P\left(C=i\right)$ denotes the prior probability of class *i*.

At this point, a QDA classifier founded on dynamic feature extraction and modified Fisher dimensionality reduction is trained.

### 3.4. Online Classification

Typically, the tasks of fault detection and fault diagnosis are carried out sequentially. For the purposes of this study, we assume that the online fault samples have already been accurately identified by the fault detection algorithm and subsequently classified using the method proposed herein.

Now, when dealing with a new pair of online samples, denoted as $x_{t}$ and $x_{t+1}$ and suspected to be faulty, the online classification stage begins by initializing them with the baseline obtained from the normal training data. Following this setup, we proceed to compute the reconstructed error, ${\tilde{e}}_{t}$ and the corresponding augmented vector, ${\tilde{x}}_{t}$, through the following equations:

(33) $$\begin{array}{cc} {\tilde{e}}_{t} & ={\tilde{x}}_{t-1}-x_{t-1}\\ \; & =x_{t}R-x_{t-1} \end{array}$$

(34) $${\tilde{x}}_{t}=\left[x_{t}\;\;{\tilde{e}}_{t}\right]$$

Following this, we project the augmented vector into a low-dimensional space utilizing the projection matrix, denoted as *W*, which has been derived from the training data for various fault classes. The calculation of the low-dimensional projection $y_{t}$ is executed as follows:

(35) $$y_{t}={\tilde{x}}_{t}W$$

Finally, we integrate $y_{t}$ into the discriminant function for each class, employing the QDA parameters obtained during the training phase as follows:

(36) $$\delta_{i}\left(y_{t}\right)=-\frac{1}{2}\log\left|\Sigma_{i}\right|-\frac{1}{2}\left(y_{t}-\mu_{i}\right)\Sigma_{i}^{-1}{\left(y_{t}-\mu_{i}\right)}^{T}+\log P\left(C=i\right)$$

For the same new sample, the highest function value designates the class with the highest posterior probability, thereby classifying the sample accordingly.

(37) $$C\left(y_{t}\right)=\underset{i}{\arg\max}\;\;\delta_{i}\left(y_{t}\right)$$

**Figure 2:**
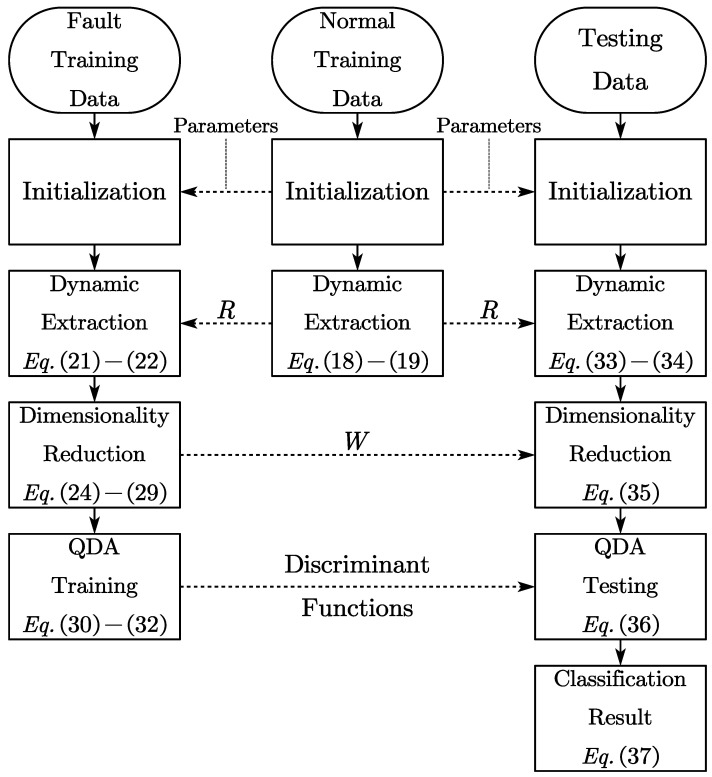
Flowchat of the proposed method.

The flowchart in Figure 2 visually outlines the methodology presented in this paper, offering a clear representation of the offline modeling and online classification procedures. It provides a clear depiction of both the offline modeling and online classification procedures, shedding light on how trained parameters are applied to newly acquired online samples.

This process commences by harnessing a substantial volume of normal process data, serving as the foundation for reference in subsequent standardization and whitening procedures. Importantly, this phase plays a pivotal role in deriving the transformation matrix *R*. Subsequently, the fault training datasets are initialized based on the normal training data. These datasets are crucial in training projection and classification models. For online samples, the process involves initialization with the normal training data as well, followed by projection and classification based on the models developed from the fault training data.

## 4. Simulation Experiment and Discussion

### 4.1. Experiment Setup in the Cold Rolling Mill Case

The cold tandem rolling process encompasses a broad spectrum of knowledge domains, including materials science, machinery, computer science and control engineering. This intricate industrial operation is marked by multi-variable coupling and relies on a range of advanced control techniques. A key parameter of concern in this process is the exit thickness of the cold-rolled strips, where the automatic gauge control (AGC) system plays a vital role in ensuring accuracy.

In the realm of AGC, addressing high-frequency faults such as servo valve gain deviations, oil contamination, pipeline leaks and displacement sensor failures is essential. Anomalies arising from these issues can propagate through the series rolling mill system, potentially causing issues like strip stacking or breakage and posing safety hazards for operators. Early detection and precise fault localization for slowly emerging issues are challenging tasks, underscoring the urgent need for an efficient AGC dynamic system fault classification method.

Given the inherent risks and destructive potential of anomalies in real rolling mills, the development of a robust simulation model is a pivotal aspect of tandem cold rolling process research. In previous work, we established a simulation model that takes into account the intricacies of the AGC system, known for its complex dynamics, nonlinearity and significant pure delay. This foundational model serves as a basis for our exploration of data-driven fault classification algorithms [43].

**Figure 3:**
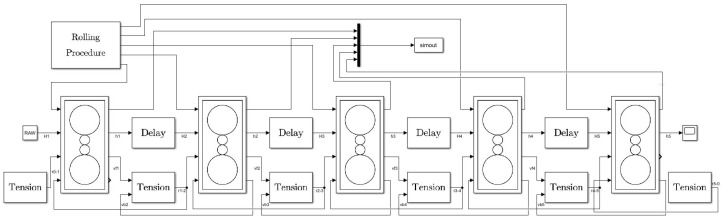
Five-stand AGC simulation model.

The five-stand AGC simulation model, as depicted in Figure 3, is a visual representation of the rolling mill modules. It not only computes rolling force and thickness but also derives strip speed through the flow rate equation, crucial for calculating the inter-stand tension. Delay modules are thoughtfully employed to facilitate the exchange of strip thickness information among the five rolling mill stand modules.

**Figure 4:**
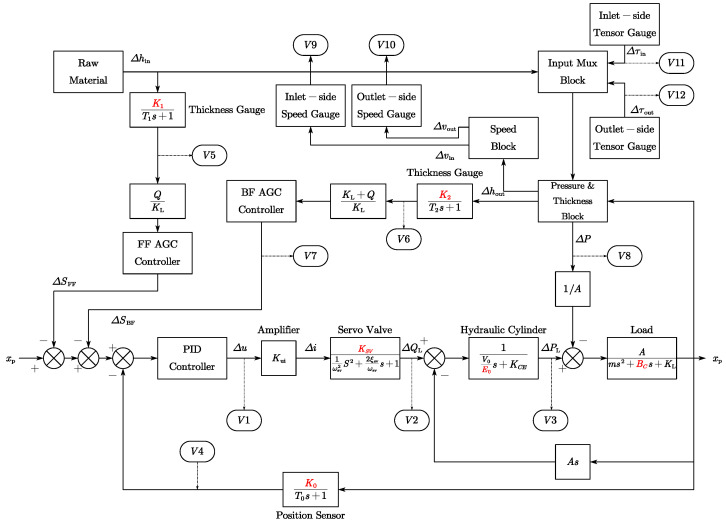
AGC control system block diagram.

Figure 4 provides a glimpse into the control block diagram within a single stand. This diagram encompasses the hydraulic position control system, along with feedforward and feedback AGC subsystems, all simulated using industry-standard mechanism models. This model’s versatility allows dynamic simulation of various variables when simulating both normal AGC loop operation and faults. Measurable variables are represented by obround blocks within the block diagram, while fault introduction points are highlighted in red font.

Table 1 presents a comprehensive inventory of measurable variables acquired from the 4th and 5th stands of the 5-stand cold rolling mill model. These variables encompass numerous critical parameters and involve data obtained from diverse sensors. The normal training data are gathered during stable operation of the simulation model, with a sampling period of 40 ms.

Table 2 provides an overview of the six distinct simulated fault classes targeting the 4th stand AGC system. Each fault within these classes has been deliberately introduced in a ramp-like progression. To thoroughly validate the algorithm’s efficacy, the simulated fault types cover various locations within the multi-loop AGC system.

To address the influence of chance outcomes, we conducted multiple Monte Carlo experiments, varying the random seeds with each run. Our experimental setup involved a normal training set comprising 10,000 samples, while the fault training set comprised 100 samples for each type, considering 24 variables derived from two stands. This configuration mirrors real scenarios, where fault history data is notably limited in comparison to normal data, while also simulating scenarios with high-dimensional features and restricted sample sizes.

All faults are introduced in ramp form, wherein following their occurrence, the respective fault points gradually increase at a slow linear rate. In the test set, the ramp slope is set at half the value of the fault training set, emulating scenarios with smaller fault magnitudes.

In our comparative experiment, we employ both LDA and QDA as control groups. To emphasize the enhancements offered by our proposed dynamic extraction method, we use the WMSD criterion as a control variable. This criterion is utilized not only by LDA but also as part of the preliminary dimensionality reduction process in QDA. This selection ensures the comprehensiveness of our experiment while providing valuable reference points. Furthermore, we introduce KFD, a widely used nonlinear FDA extension, for comparison purposes. It’s important to note that our proposed method essentially incorporates dynamic extraction as an initial step based on the foundation of WMSD-QDA.

Table 3 provides the specific parameters employed by each of these methods. In our comparative analysis, we maintain consistent parameter settings for all methods that utilize the WMSD criterion. The weight of the between-class scatter (θ) is uniformly established at 0.9. In the dynamic reconstruction phase of our proposed method, we apply a cumulative energy of singular values (CESV) set at 0.8 as the criterion for rank reduction. As for KFD, we employ a Gaussian kernel and set the bandwidth to 100n, where *n* represents the sample size. These parameter configurations have been fine-tuned to optimize the classification performance.

### 4.2. Results and Discussion

**Figure 5:**
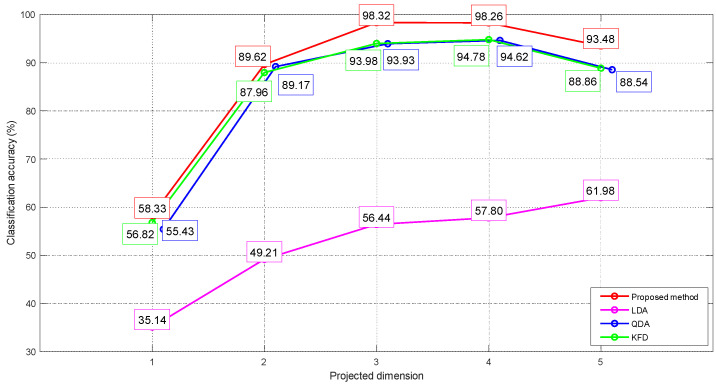
Relationship between projection dimensionality and classification accuracy.

Figure 5 illustrates our procedure for determining the optimal projection dimensionality parameter. As depicted, in the case of our proposed methods, QDA and KFD, the classification accuracy exhibits a notable increase as the projection dimensions range from 1 to 3. However, beyond this 3-dimensional threshold, the classification accuracy stabilizes, primarily influenced by random disturbances. When extended to five dimensions, corresponding to the six-class problem’s highest dimensionality, the accuracy decreases due to overfitting. Notably, although LDA exhibits a distinct trend, there’s only a marginal accuracy increase beyond the 3-dimensional projection. Consequently, to ensure uniformity and generate more comparable results, we fix the projection dimensionality at three for our comparative tests.

Table 4 presents an overview of the classification performance from 100 repeated experiments. In our analysis, we have noted that the performance of some methods exhibits substantial variability, rendering average accuracy insufficient for capturing the nuances among different algorithms. Therefore, we include the worst accuracy and standard deviation. It is essential to clarify that the “worst accuracy” in the penultimate line refers to the worst among the average accuracies for six faults over multiple experiments, rather than the worst accuracy for a single fault.

The results indicate that, in this context, LDA’s performance is notably deficient, yielding an average classification rate of approximately 50%. QDA and KFD achieve commendable average scores, hovering around 94%, yet in specific experiments, these values dip to 85%, accompanied by a considerable standard deviation. In contrast, the proposed method not only secures the highest average classification accuracy, exceeding 98%, but also elevates the lower limit of the score to more than 94%, surpassing the average scores of QDA and KFD. The significantly lower standard deviation underscores the robustness of the proposed method.

**Figure 6:**
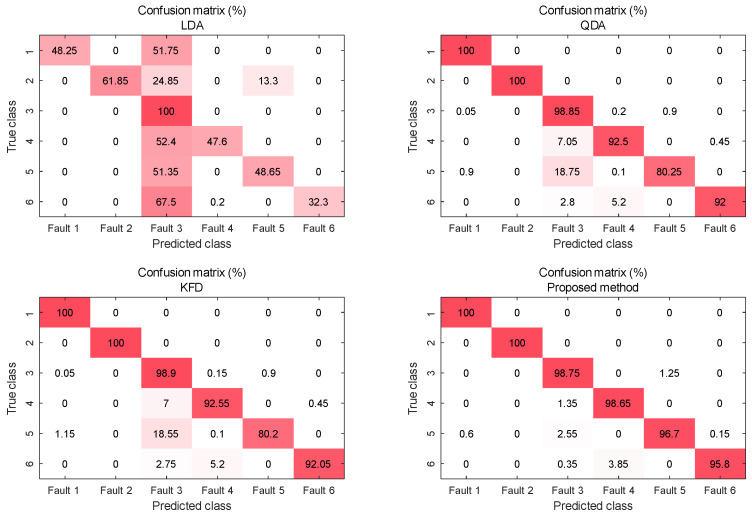
Confusion matrices of algorithms involved in the comparison.

The average accuracies individually calculated for each of the six fault classes can be analyzed by examining the first six rows of Table 4 and the confusion matrices of Figure 6.

The figure clearly illustrates a significant number of misclassifications for LDA, primarily attributing faults to fault 3, which consequently results in an overall classification accuracy of only 50%. Meanwhile, QDA and KFD exhibit similar performance to each other, effectively distinguishing faults 1 and 2. However, their ability to classify faults 5 remains limited. Their performance on fault 3 surpasses that of the proposed method, albeit at the cost of a higher misclassification rate for other fault classes.

In comparison to QDA and KFD, the proposed method offers an overall enhancement in classifying faults 3 to 6. As the potential for misclassifying other faults as fault 3 diminishes, the classification accuracy for fault 3 experiences a minor decrease but remains impressively high at 98%. For each listed fault, the proposed method consistently achieves a classification success rate of over 95%. This reconfirms the reliable performance of the proposed method in the context of this classification task.

**Figure 7:**
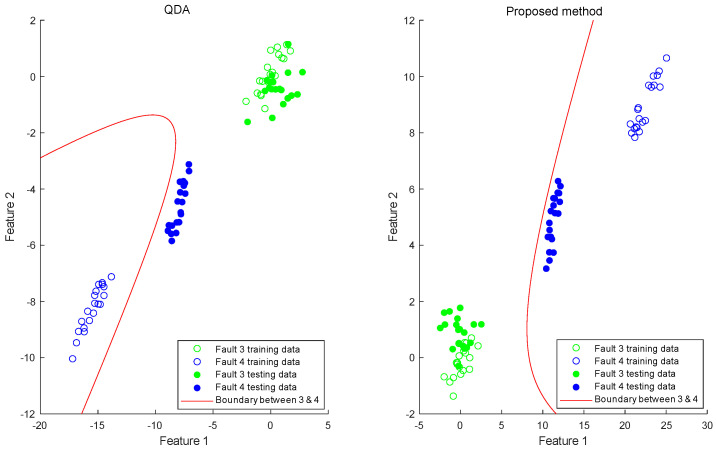
Scatter plot and discrimination boundary of faults 3 and 4.

Due to the characteristic ramp form of the faults, early samples pose greater classification challenges compared to later ones. To further elucidate the enhanced performance of early fault diagnosis through dynamic feature extraction and expansion, we focused on analyzing the initial 20 samples of faults 3 and 4 in a specific experiment, as depicted in Figure 7. In the scatter plots, the horizontal and vertical coordinates represent the top two feature directions with the highest separation after projection. The blank and filled circles respectively denote samples from the training and test sets. Additionally, green and blue colors respectively represent faults 3 and 4. The red curve outlines the quadratic discrimination boundary.

The analysis reveals that QDA performs satisfactorily in segregating the training set when the WMSD criterion is applied individually. This holds true not only for resubstitution validation but also for new samples with identical slope to the training set. However, when online samples feature smaller fault magnitudes, they tend to deviate from the classification centroid, leading to misclassifications. In contrast, our proposed method, WMSD-QDA with the inclusion of dynamic feature expansion, successfully maintains accurate early fault classification even under these conditions.

## 5. Conclusions

In conclusion, this study introduces a novel method for fault diagnosis in dynamic nonlinear systems. The main contributions of this research include the proposal of a dynamic feature extraction method and the development of an enhanced fault classification procedure. While controlling the input dimensions of QDA, this algorithm retains sufficient useful discriminant information and improves early diagnosis capabilities.

To evaluate the performance of the proposed method, a case study using a cold rolling mill system was conducted. The results indicate that the proposed method surpasses the capabilities of traditional approaches, LDA, QDA and KFD methods in terms of classification accuracy and stability in maintaining excellent diagnostic results.

While the proposed method shows promise for application in process fault diagnosis within the steel industry, challenges pertaining to real-time implementation, scalability to larger datasets and robustness across diverse fault patterns emerge as vital aspects requiring attention. Tackling these challenges is essential to advance the method’s effectiveness and ensure its successful deployment. Additionally, these areas pave the way for further exploration and refinement in our future endeavors.

## Figures and Tables

**Table 1:** Measured process variables in the simulation model for the cold rolling AGC system.

Variable	Description	Unit
V1	Output of the inner loop position controller	V
V2	No-load flow of the servo valve	m${}^{3}$/s
V3	Pressure of the hydraulic cylinder	Pa
V4	Displacement of the hydraulic cylinder	mm
V5	Strip thickness of the inlet side	mm
V6	Strip thickness of the outlet side	mm
V7	Output of the outer loop thickness controller	V
V8	Rolling force	kN
V9	Strip speed of the inlet side	m/s
V10	Strip speed of the outlet side	m/s
V11	Strip tension of the inlet side	MPa
V12	Strip tension of the outlet side	MPa

**Table 2:** Simulated fault types in the simulation model for the cold rolling AGC system.

Case	Description
F1	Change in the servo valve gain coefficient $K_{SV}$
F2	Air mixed into the oil, causing a change in the parameter $E_{0}$
F3	Change in the load damping coefficient $B_{C}$
F4	Gradual shift in the displacement sensor coefficient $K_{0}$
F5	Gradual shift in the inlet thickness sensor coefficient $K_{1}$
F6	Gradual shift in the outlet thickness sensor coefficient $K_{2}$

**Table 3:** Parameters of the algorithms involved in the comparison.

	Proposed Method	WMSD- LDA	WMSD- QDA	Kernel FDA
Parameters	θ=0.9	θ=0.9	θ=0.9	σ=100n
	CESV=0.8			

**Table 4:** Performance of the algorithms involved in the comparison.

	Proposed Method	WMSD- LDA	WMSD- QDA	Kernel FDA
Fault 1 (%)	100	48.3	100	100
Fault 2 (%)	100	61.9	100	100
Fault 3 (%)	98.8	100	98.9	98.9
Fault 4 (%)	98.7	47.6	92.5	92.6
Fault 5 (%)	96.7	48.7	80.3	80.2
Fault 6 (%)	95.8	32.3	92	92.1
Overall average (%)	98.3	56.4	93.9	94
Worst average (%)	94.8	48	84	84
Standard deviation (%)	1.48	4.55	5.02	5.12

## Data Availability

The data presented in this study are available on request from the author, H.L.
